# The role of mindfulness in stress, productivity and wellbeing of foundation year doctors: a mixed-methods feasibility study of the mindful resilience and effectiveness training programme

**DOI:** 10.1186/s12909-024-05810-7

**Published:** 2024-08-02

**Authors:** Chanais Matthias, Christopher Bu, Matt Cohen, Marc V. Jones, Jasmine H. Hearn

**Affiliations:** 1 Department of Psychology, Manchester Metropolitan University, Brooks Building, 53 Bonsall Street, M15 6GX Manchester, UK; 2 Care in Mind, Hope House, Hercules Business Park, Stockport, SK3 0UX UK; 3 North West of England School of Foundation Training & Physician Associates, Manchester, UK

**Keywords:** Mindfulness training, Foundation doctors, Junior doctors, Medical training, Meditation

## Abstract

**Background:**

Medical Foundation Year (FY) doctors demonstrate greater psychological distress compared with the general population and other student groups. This feasibility study investigated FY doctors’ perceptions of mindfulness and the impact of a mindful resilience and effectiveness training (MRET) programme on stress, wellbeing, and performance.

**Methods:**

Mixed-methods study utilising a questionnaire (study 1, *N* = 144) and a pre-post analysis design of MRET programme (study 2, *N* = 13), along with focus groups (*N* = 7).

**Results:**

In study 1 28.5% of FY’s reported using mindfulness. All five mindfulness facets were significantly, and positively, associated with mental wellbeing (*p* < 0.05). Acting with awareness (AA) and non-reactivity (NR) were significantly, positively associated with a challenge responses to stress (*p* < 0.05). Threat and loss appraisals were negatively associated with AA, NR, and non-judging (*p* < 0.01). Perceived productivity was positively associated with mindfulness facets: describing, AA, and NR (*p* < 0.001). In study 2, there were significant increases in wellbeing and mindfulness facets observing, describing, AA, and NR, and threat appraisals decreased (*p* < 0.05). The main themes identified across the focus group included Reframed Mindset, Values-Based Action, Embodied Leadership and Pedagogy.

**Conclusions:**

There exists a relationship between mindfulness, psychological wellbeing, and performance in FYs. The MRET prorgamme improved psychological wellbeing and reduced threat appraisals. Future work could focus resources on enhancing the skills of AA and NR, as this may be sufficient to bring about meaningful improvements in wellbeing, percieved productivity and cognitive reappraisal of stressful life events.

## Background

The development and maintenance of competent and compassionate doctors, requires the selection of appropriate candidates, years of medical education and ongoing protective structures across postgraduate clinical training. Unfortunately, some of the demands of medical training, including study burden, busy schedules [1] and assessment processes [2], have detrimental effects on the mental wellbeing of medical Foundation Year (FY) doctors (years 1 and 2). Research indicates that 40% of medical students and doctors reported currently suffering from depression, anxiety, burnout, stress, emotional distress, and/or another mental health condition that is impacting on their work/training/study [3], which is higher than that of the general population and age-matched peers [4]. Further, 91% of junior doctors were reportedly at high/very high risk of burnout, and reported the lowest scores for life satisfaction, happiness, and feeling that what they were doing was worthwhile [3]. More recently, 2023 research has indicated that 62% of junior doctors engaged in hazardous alcohol consumption as a coping mechanism [5]. In addition to the personal consequences of lower mental wellbeing there are also organisational (e.g., staff on sick leave) and performance consequences. For example, psychological distress may be detrimental to empathy and optimism in medical trainees [6]. Effective stress management tools and support for medical trainess, particularly in the early years of medical training, may therefore have a range of beneifts.

One approach that may be an effective stress management tool for medical trainees is mindfulness. Mindfulness is a process of bringing attention to experience life, moment-by-moment [7], with the aim to develop present-moment awareness and acceptance, rather than changing thoughts and behaviours [8]. Present-moment awareness is cultivated through attending to internal experiences such as, bodily sensations, thoughts and emotions in each moment, in a non-judgmental manner [9]. This is done through systematic training and practice, primarily through meditation, and is demonstrated to heighten self-awareness of thoughts, which helps to counter behaviours such as rumination, which often precedes depression [10]. As a result, engagement in appropriate self-care strategies and reasoned responses to day-to-day stressors can be enhanced [11].

Mindfulness-based interventions have been studied in medical students, with promising results in terms of stress reduction and improvements in wellbeing [12, 13]. For example, medical students who scored highly on a mindfulness measure, had reduced levels of the stress hormone cortisol and perceived stress levels immediately prior to exams and performed better in those exams compared to medical students with lower mindfulness scores [14]. Practicing mindfulness in this population may also enhance empathy and compassion for others [15] as it is linked to reduced rumination about the past or worry about the future [16].

Training medical students to apply mindfulness practice into their daily lives has also been found to increase performance. Mindfulness-based interventions may refine the ability to choose where to focus attention, and therefore improve performance in stressful and demanding environments [17]. To illustrate, a brief online mindfulness training programme for medical students reported improved performance, productivity, and lower perceived stress levels after completion of the programme, [16]. Currently, the literature on the relationship between mindfulness and performance is in its infancy, requiring further investigation of its relationships to mindfulness and other important outcomes in the medical profession.

For Foundation Year (FY) doctors there is a need for the development of programmes that are brief, easily embedded into clinical training, responsive to the needs and priorities of foundation year doctors and easily replicable. The current study uses the Mindful Resilience and Effectiveness Training (MRET) programme, developed by Flaxman et al. [18] who combined mindfulness with principles of Acceptance & Commitment Therapy (ACT) in order to improve well-being and performance for people in the workplace. This mixed-methods, feasibility, and evaluation study, aimed to:

1. Examine the relationships between mindfulness and key outcomes of productivity, appraisal of stressful life events, and mental wellbeing, and FY doctors’ perceptions of the utility of mindfulness in their daily lives.
2. Explore the feasibility of the MRET programme, specifically examining recruitment and retention rates due to the high time commitment required of participants.
3. Examine the utility of the MRET programme to enhance performance and psychological wellbeing in FY doctors.
4. Explore programme participants’ views of the MRET programme, its content, delivery, and development.

## Study 1 – Foundation Doctors’ Perceptions and Use of Mindfulness and is association with Wellbeing, Stress and Productivity

### Method

#### Participants

Eligible participants were recruited from the North West of England School of Foundation Training (a school of over 1,700 FY1 and FY2 doctors) across April - September 2021. Participants were required to be enrolled on a Foundation Training programme to be eligible to participate in the questionnaire study. Participation was voluntary and written informed consent was obtained from participants and any FYs who indicated they were experiencing acute stress (including from a mental health condition) were excluded from participation to minimise risk of inducing further stress and ensure that responses were not skewed by this.

Completed data were received from 144 (female *n* = 103; male = 41) participants (an 8.3% response rate). The majority of respondents were Foundation Year 1 (66.7%), with the remainder being Foundation Year 2. 72% of respondents were White/White British, Irish, European or another White background, 21.6% were Asian/Asian British Indian, Pakistani, Chinese, or from another Asian background, 5.6% were from a White and Black Caribbean, African, or Asian background and 1.4% were from a Black/Black British African background. The majority (97.9%) were employed on a full-time basis, with 2.1% employed on a part-time basis.

#### Procedure

In April to September of 2021, FY doctors were invited via email to complete an online questionnaire via Qualtrics. Invitations to complete the questionnaire were distributed via email through specific regional representatives to all FY doctors belonging to the North West of England School of Foundation Training and Physician Associates. Participants who took part in the questionnaire study gave consent before questionnaire completion. Participation was on a voluntary basis.

### Measures

#### Appraisal of life events scale [19]

The Appraisal of Life Events (ALE) scale consists of 16 items, scored on a 6-point Likert scale from 0 (not at all) to 5 (very much so). The scale includes the three primary appraisal dimensions of threat (6 items), loss (4 items) and challenge (6 items). Individuals are required to describe the most stressful event they had experienced in the last three months and to rate their response to this event on each of the 16 items. All items included in the ALE scale are positively scored with scores ranging from 0 to 30 for threat and challenge, and 0–20 for loss. In the present study the ALE subscales demonstrated acceptable internal reliability for threat (*a* = 0.607), for challenge (*a* = 0.841) and loss (*a* = 0.828).

#### Warwick Edinburgh mental-wellbeing scale [20]

The Warwick Edinburgh Mental-Wellbeing scale (WEMWBS) has 14 items, answered on a 5-point Likert scale from 1 (None of the time/rarely) to 5 (often/all of the time). The WEMWBS scale incorporates both eudaimonic and hedonic aspects of mental wellbeing. All WEMWBS items are scored positively and are summed to give a total ranging from 14 to 70, with higher scores indicating higher levels of mental well-being. The WEMWBS demonstrated an excellent level of internal reliability (*a* = 0.913).

#### Five facet mindfulness questionnaire-short form [21]

The Five Facet Mindfulness Questionnaire-Short Form (FFMQ-SF) consists of 24 items scored on five-point Likert scales ranging from 1 (never/rarely true) to 5 (very often/always true). It measures five factors representing elements of mindfulness: observing (OB) (4 items), describing (DS) (5 items), acting with awareness (AA) (5 items), non-judging (NJ) of inner experience (5 items), and non-reactivity (NR) to inner experience (5 items). Mindfulness facet scores range from 5 to 25, apart from the facet of OB, which ranges from 4 to 20. The total maximum score on the FFMQ-SF is 120, with higher scores indicating greater levels of mindfulness. The FFMQ-SF has strong psychometric characteristics, including good reliability with alpha coefficients above a = 0.70 for all facets. The FFMQ-SF demonstrated good internal reliability (*a* = 0.841) in the present study and for the five facets of OB (*a* = 0.72), DS (*a* = 0.80), AA (*a* = 0.86), NJ (*a* = 0.79) and NR (*a* = 0.82).

#### Percieved productivity

Performance of FY doctors is hard to measure given that they perform a variety of complex and demanding tasks across different teams. Due to the challenge and feasibility of selecting an independent assessor of performance we utilised a self-report measure of percieved productivity. The term productivity in this context is used to refer to performance more broadly in terms of how FY doctors feel they are performing in their role. We used a single item to measure percieved productivity which asked participants “Over the last month, roughly how productive have you felt in your job?’. Participants were required to respond using a visual analogue scale from 0–100% with 0 indicating not productive at all, and 100 indicating the most productive possible. A similar measure has been used to assess productivity in white collar workers [22].

#### Mindfulness use

Participants were asked to indicate reasons for their use or non-use of mindfulness and were able to select tick boxes to all that applied. Answer options included: to reduce stress/negative emotions, to relax, to help with sleep, and to manage pain or other physical symptoms.

#### Open text responses

Respondents were also able to respond in an open text box if they wanted to indicate other reasons for practicing mindfulness. Two further open-ended questions explored the FYs perspective of what factors might help and prevent them from engaging in a mindfulness training programme.

### Data analyses

An a priori power calculation was undertaken to establish the minimum sample size required for pearson’s correlation coefficient; based on Power = 0.80, medium effect size = 0.30, alpha = 0.05, the minimum sample size required was *n* = 85 [23]. Data were analyzed using SPSS Version 25. Data were initially examined for distribution normality and outliers. For demographic data, means and standard deviations were calculated. All data met parametric assumptions. A Pearson’s correlation coefficient was run to assess the relationship between WEMWB scale, perceived productivity, ALE scale and FFMQ-SF facets.

### Results

Data on mindfulness use showed 28.5% of participants reported using mindfulness. The most common reason for mindfulness use was to reduce stress/negative emotions (52.1%), followed by relaxation (43.8%), and managing mental health (30.6%). Other reasons indicated in open-text responses included to help with sleep (27.1%), improve concentration (18.8%) manage pain or other physical symptoms (1.4%). Reasons for not using mindfulness included not having enough time (43.1%), being easily distracted (38.2%), and/or forgetting to do mindfulness (31.3%). Some respondents (29.2%) stated they did not know how to do mindfulness, felt that it was too much effort (12.5%), did not like it (5.6%) and it made them sleepy (3.5%). The means for each scale used in the questionnaire are presented in Table 1. The most rrecent mean overall WEMWBS score for the UK population score is 51, which highlights that the FYs surveyed have a lower average of mental wellbeing (46.70; SD = 8.54) than those in who are non-medical trainees [20].

**Table 1:** Means and standard deviations of study one questionnaire measures

Scale	Mean(SD)
Warwick Edinburgh Mental Wellbeing Scale	46.70(8.54)
ALE (threat)	23.58(5.75)
ALE (loss)	12.56(5.43)
ALE (challenge)	18.36(6.43)
Five-Facet Mindfulness Scale	72.81(11.46)
FFMQ-SF (observing)	13.19(3.21)
FFMQ-SF (describing)	16.92(3.76)
FFMQ-SF (acting with awareness)	14.67(4.11)
FFMQ-SF (non-judging)	14.26(3.89)
FFMQ-SF (non-reactivity)	13.77(3.75)
Productivity	46.70(8.54)

Notes: ALE = Appraisal of life events scale, FFMQ-SF = Five Facet Mindfulness Questionnaire-Short Form

Pearsons’s correlations coefficients were calculated (Table 2) to assess the relationships between mindfulness (FFMQ-SF), mental wellbeing (WEMWB), perceived productivity and appraisal of stressful life events (ALE).

**Table 2:** FFMQ, ALE, WEMWBS and productivity correlation table

		ALE (challenge)	ALE (threat)	ALE (loss)	FFMQ-SF (Total)	FFMQ-SF (OB)	FFMQ-SF (DS)	FFMQ-SF (AA)	FFMQ-SF (NJ)	FFMQ-SF (NR)	WEMWBS	Productivity
ALE (challenge)	*r* Sig											
ALE (threat)	*r* Sig	− 0.174* 0.036										
(ALE) (loss)	*r* Sig	− 0.400** 0.000	0.448** 0.000									
FFMQ-SF (total)	*r* Sig	0.272** 0.001	− 0.318** 0.000	− 0.297** 0.000						.		
FFMQ-SF (OB)	*r* Sig	0.122 0.145	− 0.125 0.137	− 0.023 0.782	0.457** 0.001							
FFMQ-SF (DS)	*r* Sig	0.083 0.320	− 0.028 0.743	− 0.186* 0.025	0.595** 0.000	0.025 0.770						
FFMQ-SF (AA)	*r* Sig	0.193* 0.020	− 0.341** 0.000	− 0.214* 0.010	0.700** 0.000	0.202* 0.015	0.268** 0.001					
FFMQ-SF (NJ)	*r* Sig	0.154 0.066	− 0.224** 0.007	− 0.329** 0.000	0.599** 0.000	0.023 0.780	0.215* 0.010	0.294** 0.000				
FFMQ-SF (NR)	*r* Sig	0.272** 0.001	− 0.232* 0.005	− 0.126 0.133	0.682** 0.000	0.273** 0.001	0.278** 0.001	0.300** 0.000	0.236** 0.004			
WEMWBS	*r* Sig	0.308** 0.000	0.332** 0.000	− 0.413* 0.000	0.598** 0.000	0.175* 0.036	0.362** 0.000	0.475** 0.000	0.341** 0.000	0.443** 0.000		
Productivity	*r* Sig	0.230** 0.005	− 0.189* 0.023	0.252** 0.002	0.339** 0.000	0.068 0.418	0.276** 0.001	0.317** 0.000	0.129 0.125	0.221** 0.008	0.480** 0.000	

*=*p* < 0.05, **=*p* < 0.001

Notes: ALE = Appraisal of life events scale, FFMQ-SF = Five Facet Mindfulness Questionnaire-Short Form, WEMWBS = Warwick Edinburgh Mental Wellbeing Scale

#### Mindfulness and mental wellbeing

There were significant positive relationships between all five mindfulness facets and mental wellbeing: OB (*r* (142) = 0.18, *p* < 0.5, DS (*r* (142) = 0.36, *p* < 0.001), AA (*r* (142) = 0.48, *p* < 0.001), NJ (*r* (142) = 0.34, *p* < 0.001) and NR (*r* (142) = 0.44, *p* < 0.001).

#### Mindfulness and appraisals of life events

There were significant positive relationships between, challenge and two mindfulness facets: AA (*r* (142) = 0.19, *p* < 0.5,) and NR (*r* (272) = 0.15, *p* = 0.001). There were significant negative relationships between threat and mindfulness facets: AA (*r* (142) =-0.34, *p* < 0.001), NJ (*r* (142) = − 0.22, *p* < 0.5), and NR (*r* (142) = − 0.23 *p* < 0.5), and loss and mindfulness facets: DS (*r* (142) = − 0.19, *p* < 0.5), AA (*r* (142) = − 0.21, *p* < 0.5), and NJ (*r* (142) = − 0.33, *p* < 0.001).

#### Mindfulness and productivity

There were significant positive relationships between percieved productivity and three of the five mindfulness facets: DS (*r* (142) = 0.28, *p* < 0.001), AA (*r* (142) = 0.32, *p* < 0.001) and NR (*r* (142) = 0.22, *p* < 0.001).

#### Mental wellbeing, productivity and appraisals of life events

There was a significant positive relationship between mental wellbeing and perceived productivity (*r* (142) = 0.48, *p* < 001), and the challenge sub scale of the ALE (*r* (142) = 0.31, *p* < 0.001). There were significant negative relationships between mental wellbeing and the threat (*r* (142) = − 0.33, *p* < 0.001), and loss (*r* (142) = − 0.41, *p* < 0.001) sub scales of the ALE.

There was a significant positive relationship between percieved productivity and the challenge sub scale of the ALE (*r* (142) = 0.23 *p* < 0.5). There were significant negative relationships between percieved productivity and threat (*r* (142) =-0.19, *p* < 0.5), loss (*r* (142) = − 0.5, *p* < 0.5,).

Linear regression analyses were conducted to determine whether levels of mindfulness could predict ALE, mental wellbeing, and perceived productivity. The results revealed the overall model fit for all measures was significant indicating that mindfulness significantly predicts ALE [*F*(1, 142) = 4.61, *p* = 0.033], mental wellbeing [*F*(1,142) = 79.21, *p* < 001], and perceived productivity [*F*(1, 142) = 18.41, *p* < 001]. Mindfulness was also found to account for 3.1% of the variation of ALE scores, 35.8% of mental wellbeing scores, and 1.2% of perceived productivity scores. This suggests that increased levels of mindfulness can decrease negative appraisal of stressful life events, increase perceived productivity and mental wellbeing in this FY doctors.

## Study 1 discussion

Study one explored FY doctors’ perceptions of mindfulness, and the relationships between mindfulness and key outcomes of percieved productivity, appraisal of stressful life events, and mental wellbeing. The study found varying reasons FYs perceived mindfulness practice to be beneficial. Of 28.5% of participants that indicated they used mindfulness, half of them did as a strategy to reduce stress/negative emotions, closely followed by using mindfulness as a tool to relax. Moore et al. [15] found that 17% of participants engaged in mindfulness practice prior to participating in their mindfulness based 8-week programme, indicating that the sample in the present study may have been much more engaged in mindfulness for the reasons presented. In an Australian survey, 4.8% of medical students reported using alternative strategies such as meditation, sleep, or prayer as a coping strategy to deal with work-related stress [24]. In the current study, many FYs expressed not having enough time and being easily distracted as barriers to practicing mindfulness, which is has been highlighted as a concern in the literature when considering attrition and adherence to mindfulness-based interventions [25].

Specific facets of mindfulness had clear relationships with key outcomes, suggesting that these may be a particularly helpful to target in future work to enhance wellbeing, percevied productivity and threat appraisals. Our analysis showed overall mental wellbeing had the stronger relationships to mindfulness facets of non-reactivity and acting with awareness.

Non-reactivity, which refers to the ability to allow experiences to come and go without reacting to change them, had a significant and moderately positive relationship with wellbeing and a significantly negative and weak strength relationship with threat. These findings suggest that NR could be a valuable skill that enables individuals to disassociate from unhealthy coping responses [26, 27], thus buffering against negative impacts on wellbeing. Additionally mindfulness practice has also been found to enable individuals experiencing a stressful situation to focus their attention to what is currently happening and be in an accepting stance, meaning stressful situations are appraised as less threatening [28]. This perceived reduced stress can occur by changing the threat appraisals of demanding situations [28], which could explain the negative relationship between mindfulness and threat and the positive relationship between mindfulness and wellbeing. See the general discussion section for further consideration of the relationships established in study one.

## Study 2 – MRET feasibility study

### Method

#### Design

A pre-post analysis intervention study with within-groups comparisons (participants completed outcome measures at baseline and upon completion of the MRET programme), followed by focus groups at completion of the programme and again at 3 months. As this was a feasibility study with a focus on the development of the programme, no control group was employed.

#### Participants

Twenty FY doctors enrolled to take part in the MRET programme. The programme capacity was limited to twenty following the mindfulness teachers’ judgement of the largest class size they could suffcienty interact adequately with. Overall, 13 FY doctors took part in the MRET programme for the full four weeks (11 females, two males), and completed all measures, indicating a drop-out rate of 35% (i.e. 7 of the original 20 who enrolled onto the programme dropped out). Of the 13 participants who completed the MRET programme, four were FY1 and nine were FY2. Seven FY doctors took part in the post-programme focus group and four in the 3-month post-programme focus group. Twelve participants indicated they were in full-time employment, one worked part-time or less than full time. Over half of participants (53.9%) on the programme were White/ White British, Irish, European or another White background, 15.4% were Asian/Asian British Pakistani or Chinese, 7.7% identified as White and Asian and 7.7% as Black/Black British African.

### Procedure

#### Recruitment

Recruitment for the programme took place from 30th March – 27th April 2021. Programme adverts containing a link to register interest were distributed to foundation doctors in the North West Foundation School via emails sent by each trust foundation programme director. Adverts were also delivered via communication with the Nort West foundation doctor board, a group of representative foundation year 1 and year 2 doctors. Participants were selected for the programme on a first come first served basis. Ninety-four FY doctors registered interest for the 20-person programme.

#### Programme facilitators

Two teachers who had a medical background as practicing consultant doctors delivered the MRET programme. Both attended the MRET programme as a participant first, before attending two group training days, and both had significant prior experience of teaching mindfuless. Additional development days were run to support with teaching the programme online, and supervision was provided to the teachers throughout their training and delivery of the programme to FY doctors.

#### Programme procedure

All participants were required to complete the pre-programme questionnaire. The questionnaire included demographic items, a perceived productivity item, the WEMWBS, ALE Scale and the FFMQ-SF (as described in study one). Once the programme was completed, the same questionnaire was sent to participants via a Qualtrics survey link for completion.

The programme took place in May 2021, with four 2-hr sessions delivered weekly online via Zoom (due to social distancing requirments in places during the COVID-19 pandemic) for four weeks. In advance of the first session participants were sent an email containing the programme handbook and a link to join the weekly sessions.

Sessions involved examination of the principles of mindfulness, group discussions and teacher-led activities. Participants were guided through short mindfulness practices and taught how to consider self-care using value-based prioritization (see Table 3 for programme overview). For participants to receive a certificate of programme completion they were required to attend a minimum of three out of the four sessions.

**Table 3:** Overview of the MRET programme

Week	Content
1	*Workshop activities* • Introduction to the programme. • Breakout room: meet and greet, and reflection on activities performed in autopilot vs. those with full awareness. • Teaching: the three pillars of psychological wellbeing (OPEN, AWARE & ACTIVE) • Mindful activity: mindful eating exercise • Teaching: introduction to ACTIVE pillar and the concept of value-based actions - identifying behaviours that express a personal value • Teaching: introduction to OPEN pillar of how to skilfully and non-judgementally relate to inner experience using passengers on the bus analogy.
	*Home practice* • Mindful activity: perform a routine activity with full awareness • Meditation: daily practice – 5 + minutes • Values based action: perform an activity that helps express an identified personal value
2	*Workshop activities* • Meditation: the body and breath • Teaching: introduction to the thinking and sensing modes • Breakout room: reflection on participants experiences of the value-based action homework • Teaching: internal barriers to values-based action and techniques to manage internal thoughts or feelings (OPEN pillar)
	*Home practice* • Mindful activity: perform a routine activity (different to week 1) with full awareness • Meditation: daily practice − 10 min • Values based action: identify promoting and challenging factors to value-based actions.
3	*Workshop activities* • Meditation: body scan • Breakout room: reflection on home practice • Activity: identifying specific value-based actions for work, health, relationships & leisure domains • Mindful activity: physicalising activity to illustrate the space between the self, thoughts, and feelings
	*Home practice* • Mindful activity: perform a routine activity (different to weeks 1 & 2) with full awareness • Meditation micro-practice: the 3-step breathing technique • Values based action: identify promoting and challenging factors to value-based actions.
4	*Workshop activities* • Meditation: hearing based meditation • Activity: passengers on the bus to develop techniques to skilfully relate to inner experience • Breakout room: reflection on participants experiences of the value-based action activity • Meditation: 3 step breathing technique

#### Focus groups

Participants completing the MRET programme were invited to take part in post-programme focus groups upon completion of the progamme, which aimed to explore participants views of the MRET programme, its content, delivery and development. All focus groups were conducted remotely by CM and JH, lasted approximately 60 min, and were recorded and transcribed verbatim. At the beginning of each focus group verbal consent was taken and audio-recorded before beginning the discussion. The questions were in a semi-structured format, with a topic guide to aid discussion in areas participants may have addressed.

#### Data analyses

When examining the programme data, the same process was implemented as outlined in study one, to generate scores for each participant who completed the pre-post programme questionnaire. The data met all parametric assumptions. Paired samples t-tests assessed pre-post changes in mental well-being, appraisal of life events (challenge, threat and loss), mindfulness (OB, DS, AA, NJ and NR) and percieved productivity.

Focus group data was analysed after all focus groups had been completed, using inductive reflexive thematic analysis as described by Braun and Clarke [29, 30] Analysis was supported by NVivo 12 software, enabling a thorough data-management process, enhancing the transparecny of findings. Analysis was initiated by CM, who initially read each transcript twice to become familiar with the data. CM performed line-by-line coding and initial findings were triangulated with CB and MC, who systematically added a commentary from a medical perspective to ensure credibility and dependability. CB and MC provided contextual information to participant quotes to ensure meaning was retained during coding. The process of coding and theme development integrated descriptive (what the participants said) and interpretive elements (the researcher’s subjectivity) to consider patterns not so obvious [29].

Following this, codes were collated into relevant themes, which involved grouping together common patterns of shared meaning, the central organising concept of RTA [30]. All codes and themes were reviewed and refined by all authors several times as part of the analytical process, which required re-reading all codes that had been designated to a theme, to determine whether a meaningful pattern was evident. The purposes of refining the codes and themes was to ensure analysis captured the nuances of participants’ responses. This iterative process allowed the authors to develop a deeper understanding of the data, resulting in clearer codes and themes that better represented the perspectives of participants.

The writing up phase enabled for any themes that did not seem to ‘fit’ to be adjusted accordingly and the final thematic structure was achieved. Transcripts were re-read by CM, CB and MC to verify that all the themes mapped onto the study aims (exploring participants’ views of the MRET programme, its content, delivery and development). Writing up the themes, involved selecting extracts from the data for each with accompanying narrative that highlighted the views of the participants. All authors discussed and agreed the final themes.

### Results

Data were included for the 13 participants who completed the four-week MRET programme (4 attended all four sessions, 3 attended three sessions) and provided all data points. Of those who dropped out, 6 did not complete the programme, and 1 completed the programme but did not complete baseline data. Prior to participating on the programme, only 23% of participants indicated that they practiced mindfulness regularly compared to 69% post programme. The pre- and post-programme data are reported in Table 4.

**Table 4:** Means, SDs and paired sample t-test result for pre- and post MRET programme scores (*n* = 13)

Measure	Pre-MRET Mean (SD)	Post-MRET Mean (SD)	Paired difference (SD)	t-value	Sig.
WEMWBS	41.08 (8.48)	47.69 (7.77)	-6.62 (6.68)	-3.572	0.004*
ALE (challenge)	16.85 (5.01)	16.92 (3.48)	-0.077 (4.83)	-0.057	0.955
ALE (threat)	28.08 (4.15)	22.08 (8.07)	6.00 (9.29)	2.328	. 038*
ALE (loss)	14.23 (5.34)	13.00 (5.46)	1.23 (4.36)	1.017	0.329
FFMQ-SF (total)	45.00 (12.70)	52.00 (13.00)	-9.85 (11.53)	-3.079	0.010*
FFMSQ (OB)	12.00 (3.74)	14.31 (1.93)	-2.31 (3.71)	-2.245	0.044*
FFMSQ (DS)	15.15 (3.83)	17.31 (3.12)	-2.15 (3.21)	-2.419	0.032*
FFMSQ (AA)	12.15 (3.72)	15.31 (3.52)	-3.15 (4.10)	-2.774	0.017*
FFMSQ (NJ)	13.54 (5.17)	13.23 (4.55)	0.31 (4.37)	0.254	0.804
FFMSQ (NR)	11.31 (2.29)	13.85 (3.69)	-2.54 (3.67)	-2.497	0.028*
Productivity (%)	62.92 (11.70)	71.46 (12.27)	-8.54 (14.48)	-2.126	0.055

*p < 0.05

Notes: ALE = Appraisal of life events scale, FFMQ-SF = Five Facet Mindfulness Questionnaire-Short Form, OB = observing, DS = describing, AA = acting with awareness, NJ = non-judging, NR = non-reactivity, WEMWBS = Warwick Edinburgh Mental Wellbeing Scale

From pre- to post-programme, significant increases were demonstrated in overall mindfulness (*p* < 0.05), as well as individual facets of OB (*p* < 0.05), DS (*p* < 0.05), AA (*p* < 0.05), and NR (*p* < 0.05). Participants reported significantly higher levels of mental wellbeing post-programme compared to at baseline (*p* < 0.05). Threat apprisals also significantly decreased from pre to post-programme (*p* < 0.05). Mean productivity ratings also increased from pre- (62.92%, SD = 11.70) to post- programme (71.46%, SD = 12.27), however this difference was non-significant *(p* = 0.055).

### Focus group results

In total, seven FY Doctors participated in two focus groups; seven participated in the post-programme focus group and four of them participated in the second focus group 3 months later. Across the two focus groups, three key themes were identified; Reframed Mindset, Values-Based Action, Embodied Leadership and Pedagogy.

#### Theme 1. Reframed mindset

At the first post-MRET programme focus group, participants considered that increased awareness of theirs and others’ emotions through their improved mindfulness consequently improved their interactions in the workplace:

*“I think obviously being mindful and aware of your own emotions helps when interacting with anybody*,* not just patients because you can be aware of how their emotions are affecting you and vice versa”.*

This gives some indication of how the programme impacted doctors internally, but also that some participants felt mindfulness practice affected their external behaviour via increased awareness during interactions with patients or colleagues. Likewise, all participants reflected that since completing the MRET programme, they were thinking more frequently about mindfulness, with many adopting a new perspective on how mindfulness practice can be integrated into their day:

*“I had a few experiences with it crossing my mind*,* now would be a time where I should really use mindfulness. And it’s not always practical*,* in the middle of a working day always to be like*,* hold up*,* you know*,* sorry*,* I’m just gonna exit the ward (laughter)*,* I’ll be back in ten… Recognising it*,* even if you can’t then step out*,* meditate*,* or do whatever else activity in that moment…it’s still mindful. So actually*,* I think it’s positive*,* even if you don’t then go on to do an activity”.*

All participants acknowledged being more self-aware during times of increased stress, specifically to both their mind and body. This demonstrates how the programme content translates into behaviours, with them now engaging in short breaks, or ‘micro-practices’, during their working day. This is a key consideration to recognise, given that busy schedules of FY doctors may prevent them from being able to engage in longer practices sometimes advocated in mindfulness training.

*“Towards the latest sessions, when I was a bit more understanding of how to do the mindfulness exercises, I used the time like, when I’m on A&E now, if I have a half an hour break, I’d spend maybe 10 min in that break doing an exercise. And I did feel like that created a gap mentally between the patients that I’ve just seen and the patients I was going to see.”*

At 3-months post-MRET programme, micro-practices were still being implemented by FY Doctors during stressful periods at work to create space between clinical duties such as seeing patients or taking referrals. With this reframed mindset of allowing oneself to use these ‘micro-practices’, FYs felt able to ‘punctuate’ their working day by utilising mindfulness in these gaps to create psychological space:

*“You know*,* being just able to be aware that you know you’re under stress*,* there’s a lot going on. You maybe have fifty million things to do*,* but acknowledging it*,* being aware of it and having you know some small techniques as was said before*,* just take 2 minutes to yourself. Go for a quick water break or toilet break. Take the time to actually settle yourself down. Reset and then come back with a fresher mindset. So that little bit of time actually gives you back quite a lot (.) to kind of de-stress and put your mind together”.*

Participants also discussed examples of where they could go to take short breaks during times of increased stress at work, with one suggesting a toilet break as a way to step away. This provided a ‘safe space’ in a high-pressured hospital setting, making it easier for participants to digest the ideology of ‘micro-practices’ ‘in the context of taking a 2-minute toilet or water break. This highlights how the programme elicited meaningful psychological and behavoiural change within the participants.

#### Theme 2. Values-based action

Participants identified learning about and integrating values-based action as crucial components of mindfulness and that this learning was built up across the whole programme:

*“The fact that it wasn’t solely just*,* we’re going to meditate now*,* and we’re gonna do a different meditation. It was like value-based learning*,* and then the activities built on each other each week like I personally thought that was quite nice”.*

This progressive learning also fed into supporting course participants to identify and attend to their personal and professional values as well as an intention to commit to those values, despite difficulties they might encounter in their day-to-day work:

*“Identifying core values. This is something to always go back to when making decisions and considering mindfulness*”.

Participants recognition of their own values helped to inform their values-based action and fostered flexibility, awareness, regulation, connection and optimism. This meant participants felt better able to recognise the moment of choice that occurred when they were emotionally triggered and subsequently choose values-based behavior over emotion-driven urges. In particular, this was commented on as an element of the programme that could facilitate long-term psychological and behavioural change:

*“I also really like the values based for me I kind of feel like …throughout all of the sessions was more of a like… here’s a long term approach that we can take up which was very nice*,* and it was nice to do something that was kind of well thought not just for work*,* but for like personal life*,* but that wasn’t like here*,* you can do this in your extra time and it’s a lecture about this medical condition and how to treat it (Laughter)”.*

Significantly, the recognition of one’s values and engagement in values-based action demonstrated the effectiveness of the programme in supporting participants to understand their personal priorities. This brought benefits in terms of stress and wellbeing that reached beyond the workplace and into personal life, bringing meaning and vitality to daily functioning.

#### Theme 3. Embodied leadership and pedagogy

All participants reflected on the pedagogical approaches used within the programme to support understanding, skills development, and implementation of skills in daily life. In particular, the leadership of the programme by teachers who embodied mindfulness in their own lives and in the facilitation of the course were a particular strength. Participants appreciated that reflective activities and discussions were embedded in the course, in which personal experiences were shared and seen as normalising:

*“I liked that we talked in small groups about what we thought about the exercise and stuff. I think people brought different insights and it was nice having that in a small group then bringing it back because I think sometimes people share more”.*

The comments highlight feelings of comradery and connection, creating a sense of shared understanding of one another’s experiences within the safe space of the programme discussions. This was also reinforced through the role of the programme teachers, also in the medical profession, who modelled how mindfulness can feature in the work environment:

*“I think for me it was the tutors*,* as they seemed like they knew what they were doing*,* they’ve got a good background of it. And I was like*,* you know*,* what these are trusted people. So*,* like*,* I think we’re in good hands here and for me like*,* that would have made me turn off at the first session if I just felt like*,* they weren’t like*,* good people or didn’t have (.) didn’t know what they’re talking about*,* basically”.*

Participants were validated by the teachers who acknowledged the challenges of medical training and practice, especially during the foundation years, adapting to a forever changing, stressful environment, whilst managing multiple tasks at work. Likewise, the ways in which the teachers personally embodied mindfulness were reflected upon:

*“I thought that [names of teachers] were like*,* just calm*,* (.) quite calm presences. And I thought that*,* particularly that [teacher name] was like*,* very just very*,* like*,* somebody who delivered something very calmly*,* and maybe that’s partly because her background is in psych or*,* but just I really enjoyed the delivery. I enjoyed listening to them in terms of their like delivery*,* and they’re speaking and the like*,* feeling that they created*,* I think they were like*,* they were perfect for the role”.*

This embodiment of mindfulness served to reinforce participant perceptions of its value and role within medical training and practice, and served to motivate engagement in the programme each week. However, some participants commented on feelings of repetitiveness of the programme:

*“What I found harder to engage with was*,* at times it felt very repetitive. And I know repetition is important for creating a habit but also when you’ve been at work for eight hours and then you sit there and try and listen to something that you’ve listened to before when it’s eight o’clock in the night*,* I found it quite hard if it was something I’d heard the two previous weeks”.*

Given the nature of mindfulness training programmes that require repeated engagement in mindfulness exercises, this is perhaps unsurprising. Importantly, participants recognised the value in this repetitiveness in terms of creating habits, but present important considerations for maintaining engagement and motivation in future work.

## Study 2 discussion

Study two aimed to investigate the feasibility and utility of a brief four-week mindfulness training programme for FY doctors and to explore their experiences of the programme. Upon completion of the four week brief MRET programme, more FYs reported using mindfulness than at baseline, indicating the programme was efficacious in initiating or increasing their use of mindfulness. FYs reported significantly increased levels of mindfulness across four facets (OB, DS, AA, NR), mental wellbeing, and perceived productivity, and were less likely to appriase stressful events as threatning post-programme.

Findings indicated significant improvements in wellbeing, elements of appraisals of life events, mindfulness, and perceived productivity, demonstrating the programme’s success in initiating beneficial change in key outcomes of importance to FYs, consistent with previous studies that show mindfulness training to be useful across populations [13–15]. This study has illustrated that mindfulness training has the ability to ehance different skills associated with different facets of mindfulness. Therefore, the change desired by engaging in mindfulness training may influence which mindfulness skills take priority for development in programmes such as MRET. For example, acting with awareness appears to be strongly related to perceived productivity, wellbeing and challenge appraisals, suggesting that this could be a target for focused minfulness training/interventions. These findings were also reinforced by the qualitative themes around reframed mindset and values-based action.

## General discussion

This paper presents a novel, mixed-methods approach to understanding the role of mindfulness in wellbeing, cognitive appraisal of life events and perceived productivity of FY doctors within the NHS. Study one demonstrated clear relationships between mindfulness and wellbeing, cognitive appraisal of life events and perceived productivity, with key elements of mindfulness (acting with awareness, non-judging, and non-reactivity) being particularly strongly associated with these measures. Notably and significantly, there are specific elements of mindfulness that could be helpful for different outcomes which should be examined in further detail in future, a novel finding in this study. As such, these elements may be a particularly helpful to target in future work to enhance wellbeing, productivity, and threat appraisals.

The ability to act with awareness (AA) was the only facet of mindfulness significantly associated with every variable in study one. For instance, AA was positively associated with wellbeing, perceived productivity and challenge appraisals, and negatively associated with threat and loss appraisals. This increase of awareness comprises a fundamental aspect of the empirical evidence for mindfulness training, and these findings are consistent with the literature [31–33]. Given that FYs in this study indicated feeling less than 50% productive in their job at the time of completing the survey, this is an important finding, suggesting that future research and training programmes could focus resources on enhancing the skill of AA in particular. This may be sufficient to bring about meaningful improvements in wellbeing, productivity and cognitive reappraisal of stressful life events.

The ability to non-judgmentally observe and stay calm with arising thoughts and emotions is a key factor in successful emotion regulation [34], and was negatively associated with loss, threat, and positively associated with mental wellbeing in this study. This implies that participants were better able to examine and engage with their emotions effectively, soon after an emotion occurred. The ability to regulate emotion in this way has been associated with a range of thought action repertoires and more positive states of mind [35, 36]. Given that the average wellbeing score was 5-mean points lower in this study sample than that of general population samples in the UK (indicating a lower wellbeing than the general UK population; 20), this may be another key element of mindfulness training that could be focused on to enhance outcomes in future for FYs.

Likewise, the ability to observe one’s thoughts and experiences in a non-reactive manner (NR) was positively associated with appraisal of challenge and negatively associated with the appraisal of threat, consistent with the current literature which suggests mindfulness reduces stress by changing the threat appraisals of demanding situations [28]. Mindfulness practice has beneficial effects for individuals when experiencing a stressful situation; when individuals focus their attention to what is currently happening and are in an accepting stance, stressful situations are appraised as less threatening [28]. Similarly, as scores for NR increased, so did levels of mental well-being and perceived productivity in this population, comparable to systematic review evidence that found when General Practitioners (GPs) completed mindfulness-based interventions, positive effects on psychological wellbeing and occupational wellbeing were observed in terms of decreased chances of burnout, increased levels of work engagement [32]. This demonstrates further specific avenues of mindfulness research to explore to ensure that key elements of mindfulness associated with positive change are examined and enhanced further.

Study two found a brief 4-week mindfulness training programme to be a feasible and acceptable addition to FY doctors’ repertoire of skills. The self-reported improvements to mental wellbeing, mindfulness, perceived productivity, and cognitive appraisal of stressful life events suggests that programmes such as this could be a feasible and somewhat efficacious method of reducing stress and burnout among this population. In particular, the shorter length of the course, leadership by mindfulness teachers in the medical profession, shorter mindfulness practices, and focus on core values were seen as strengths of the MRET programme.

Valuable messages were identified from the focus groups, including a new or reframed mindset and perspective they felt they had developed as a result of participating on the programme, along with a clear appreciation and integration of values-based action. Many discussed being more self-aware, consistent with evidence in the literature looking at outcomes of mindfulness training [15]. Importantly, this led participants to engage in micro-practices of mindfulness techniques learned during the sessions and is an important demonstration of enhanced psychological flexibility, a process based in the interaction of cognition and direct environmental contingencies that allows a person’s behavior to persist or change in line with their long term goals and values [37]. This could be an innovative way forward for mindfulness-based interventions, as short ‘bursts’ of mindfulness practice (micro-practices) may be more feasible to adhere to and implement into a busy day at work for FY doctors.

Programme pedagogy was another key theme identified with participants relishing the diversity of teaching methods used on the MRET programme to help facilitate their learning and skills development, which took their learning beyond the association of mindfulness being perceived simplistically as ‘just’ meditation [38]. Likewise, there was recognition of the comradery and community generated among the participants. The literature highlights the benefit of programmes such as this, particularly for medical trainees as it has been found to make the stress of medical school more manageable, providing individuals with opportunity to engage with self-care and promoting openness toward mind-body skills [39]. Being able to discuss and reflect on stressful experiences with individuals with shared experience served to motivate continued engagement in the programme. This has been demonstrated across different populations including nurses and police officers with participants feeling more open around their peers as a result of mindfulness with indiviudals in their profession [40, 41].

The results from both studies suggest that psychological improvements can be achieved through applied mindfulness in a population of FY doctors. The current literature describes difficulties with attrition and adherence to mindfulness-based interventions in this population [25] thus, participation in a 4-week brief mindfulness intervention would be of value to FY doctors given their demanding schedules. However, it is worth noting that the 35% drop-out rate in the current study is considerable and barriers to engagement should be investigated further to enhance engagement in future interventions. Both studies support the suggestion that the integration of mindfulness training as a supportive mechanism in medical training is warranted and of value, although currently the United Kingdom’s General Medical Council [42] currently only advocates offering mindfulness to medical students rather than FY doctors. Indeed, multiple medical schools already provide mindfulness resources to support their students (11; 43). Others have furthered this by integrating mindfulness into their curriculum, such as Monash University in Australia, with studies showing decreased depression and hostility, even pre-exam time, as a result [12].

This study highlights that mindfulness training may be an innovative way to increase levels of wellbeing, cognitive reappraisal of stress, and perceived productivity in FY doctors. Implementing applied mindfulness practice could act as a buffer against some of the risks associated with the being a medical practitioner with regards to stress, burnout, medical errors, malpractice, depression, and reduced productivity [13] and the present study provides a foundation upon which mindfulness-based interventions could build on in future work to enhance the wellbeing of the medical workforce.

### Limitations and future directions

Within study 1, confounding factors may have impacted the findings. Specifically, whilst the inclusion criteria excluded people experiencing acute stress from participating, they may still have taken part in the study. Likewise, other factors, such as presence of specific mental health conditions, or prior experience of training to support mental health, stress, or resilience in the workplace were not accounted for within the analysis, and need to be considered when interpreting the study findings. Finally, it was not possible to ascertain exactly how many FY doctors the questionnaire was sent to within study 1, and we are therefore unable to report the overall response rate.

The intention-behaviour gap refers to the relationship between an individual’s intentions to perform a particular behaviour and actual behavioural engagement [44]. Whilst in study two, some FYs expressed that values-based action helped them enact mindfulness practices between sessions, this was not always sufficient for FYs to create a habit and sustain the potentially long-term benefits of mindfulness. This was similar to findings reported in Verweij et al. [45] where residents reported improved awreness and insight but no changes in attitude or behvaiour.

The behaviour gap can be alleviated by identifying implementation intentions and action planning, two techniques frequently adopted to bring about health behvaiour change [46]. By increasing the contextuality of the intention and identifying what (the intention is and any facilitators to help achieve the intention) when (time), where (location), how (steps, tools, strategies) the behaviour (mindfulness practice) will be completed. Considering setbacks and/or challenges to practice and how these will be overcome using stratgies learned during the mindfulness programme would be useful, as would a set date to review these plans. This specific type of action planning could increase adherence to mindfulness practice over time, which is congruent with the goals of the participants as indicated during the focus groups. Further work is required to explore the efficacy of implementing these novel suggestions into mindfulness-based interventions.

The dropout rate for study two was 35% with FYs reporting reasons such as work shifts clashing with the programme, attrition and adherence, all of which are previously reported as challenges within this population [25]. It would be beneficial for future studies to examine barriers faced by FYs completing mindfulness training and how they could be overcome. Additionally there is a selection bias which may have reduced the drop out rate since participants secured their place for MRET programme on a first-come first-served basis hence may be more motivated than other cohorts of foundation doctors. Likewise, implementing the programme with a larger and more gender-diverse sample would be needed to draw conclusions that can be generalised to the wider medical trainee population, given that gender may moderate the effect of mindfulness [47]. Longitudinal work would also be benefical in establishing the extent to which the programme can sustain beneficial change given that post-intervention outcomes were only assessed directly following the MRET programme. Finally as both studies took place across the covid-19 pandemic this may have infuenced participants perceptions of stress and affected adherence to an evening course.

## Data Availability

The datasets generated and/or analysed during the current study are not publicly available but are available from the corresponding author on reasonable request.

## Abbreviations

WEMWBS: Warwick Edinburgh Mental Well-being Scale
ALE: Appraisal of Life Events Scale
FFMQ: Five Facet Mindfulness Questionnaire
FFMQ: SF-Five Facet Mindfulness Questionnaire-Short Form
FY: Foundation Year Doctors
MRET: Mindful Resilience and Effectiveness Training
OB: Observing
DS: Describing
AA: Acting with Awareness
NR: Non-reactivity
NJ: Non-judging
